# HEMS and the challenges of an aging population: a nationwide study of interventions

**DOI:** 10.3389/fpubh.2026.1746463

**Published:** 2026-02-11

**Authors:** Łukasz Czyżewski, Łukasz Dudziński, Andrzej Silczuk, Marcin Podgórski, Patryk Rzońca, Pandur Attila

**Affiliations:** 1 Department of Geriatric Nursing, Medical University of Warsaw, Warsaw, Poland; 2 Department of Medical Rescue, Medical University of Warsaw, Warsaw, Poland; 3 Department of Community Psychiatry, Medical University of Warsaw, Warsaw, Poland; 4 Department of Human Anatomy, Medical University of Warsaw, Warsaw, Poland; 5 Department of Oxyology and Emergency Care, Institute of Emergency Care, Pedagogy of Health and Nursing Sciences, Faculty of Health Sciences, University of Pecs, Pécs, Hungary

**Keywords:** geriatrics, helicopter emergency medical services, HEMS, older adults, prehospital care

## Abstract

**Purpose:**

We aimed to characterize HEMS missions in Poland in 2015–2024 among patients aged ≥65 years and to describe unadjusted associations between age strata and escalation of advanced interventions as well as on-scene mortality after HEMS arrival.

**Materials and methods:**

We performed a retrospective analysis of the national HEMS registry in 2015–2024, including patients aged ≥65 years. Diagnoses were grouped into five clinical domains. The primary endpoint was on-scene mortality after HEMS arrival (death on scene recorded as mission outcome left on scene - death); dispatches coded as dead on arrival (DOA) were excluded. Secondary endpoints were use of advanced airway management, Prehospital Emergency Anaesthesia (PHEA), and mission outcome. We used univariable logistic regression to estimate unadjusted odds ratios (OR) with 95% confidence intervals (95% CI) for each exposure - outcome association.

**Results:**

Among 30,075 missions, neurological (42.8%) and cardiovascular (31.0%) conditions predominated. Most missions occurred in daytime (92.5%) and in rural areas (57.1%). Compared with the 65–74 group, patients aged ≥75 years less often required escalation to advanced interventions, including intubation (OR 0.71; 95% CI 0.66–0.76), PHEA-consistent sedation (0.76; 0.71–0.80), and neuromuscular blockade (0.66; 0.61–0.72). They also had lower on-scene mortality (0.75; 0.69–0.83). The highest on-scene mortality risk occurred in the cardiovascular domain (OR 6.20; 4.93–7.80).

**Conclusion:**

In Poland, HEMS provides critical access to rapid, advanced prehospital care for older adults, especially in rural regions. The observed associations highlight age-stratified differences in intervention intensity and on-scene mortality; however, results are unadjusted and may be influenced by differences in case-mix, illness severity, and treatment limitation decisions. Frailty was not measured in the registry; therefore, any interpretation in terms of frailty should be considered hypothesis-generating and requires prospective studies incorporating standardized frailty instruments (e.g., CFS).

## Introduction

Poland’s national Emergency Medical Services (EMS) play a pivotal role in public safety. The system comprises field EMS teams and hospital emergency departments (EDs). Field teams operate as ground ambulances and as air units based at Helicopter Emergency Medical Service (HEMS) bases (1–3). HEMS delivers prehospital care and conducts medical rescue flights using the air route. Missions include responses to acute illness and trauma, interfacility transfers (IFT), and international medical transport. Because our primary aim is a nationwide characterization of HEMS workload and advanced prehospital interventions in older adults, we retained both primary missions and IFT in the overall cohort as complementary components of real-world HEMS activity. Polish HEMS also supports the transport of blood, medical devices, medicinal products, and transplant-related consignments (4, 5). In older adults, HEMS offers added value beyond shorter time to first intervention. Early oxygen therapy, analgesia, and cardio-respiratory stabilization are combined with accurate destination triage to a facility matched to the presumptive diagnosis, for example a stroke center, a cardiac catheterization laboratory, or an ED. The HEMS profile of Advanced Life Support (ALS) interventions, including endotracheal intubation, mechanical ventilation, sedation, and vascular access, may reduce secondary injury and iatrogenesis caused by procedural delays. Prehospital decisions in patients aged 65 years and older are shaped by baseline vulnerability, comorbidity burden, functional status, and goals of care, in addition to the acute clinical presentation. Although frailty instruments have been proposed for use in prehospital settings, frailty was not measured in our registry and is therefore not evaluated in the present study (6, 7).

Population aging is a global phenomenon that has accelerated in recent decades. Poland, similar to other high-income European Union countries, will increasingly experience its consequences. Rising life expectancy and the growing share of older adults reflect advances in medicine, better health literacy and lifestyle modification, improved hygiene, and wide preventive programs such as vaccination and screening. EUROSTAT data indicate that seniors now constitute about 20% of Poland’s population, with further growth expected (8–10). The World Health Organization (WHO) commonly defines “older persons” as those aged 60 years and above; in clinical research, the 65 + threshold is also widely used. Current projections show a rapid rise in the proportion of older adults in Poland, which will increase demand for prehospital interventions and specialized care pathways (8–10). At the same time, the distribution of the population and medical infrastructure creates unequal access to EDs and procedural centers. In this context, HEMS becomes a key equalizer by shortening time to treatment through rapid triage and direct transport to referral centers while maintaining high patient safety standards (11).

To mitigate or slow population aging and to ensure adequate care for seniors, the Polish government has introduced multiple legislative and regulatory initiatives. The challenges of an aging society also drive scientific analyses and institutional strategies (12, 13).

The PolSenior study, one of several initiatives focused on the 65 + population, defines the care needs of older adults, including health, social, and economic costs, the role of family, institutional solutions, and long-term care. In this area, the government develops and implements regulations within senior policy. The goals are to ensure care, dignified living conditions, medical support, and quality of life appropriate to age and to deficits arising from chronic diseases and injuries (14–16).

Health risks in geriatric patients include physical and psychological burdens that markedly impair function. After age 65, common problems are dementias, late-life depression, urinary and fecal incontinence, hearing and vision impairment, disordered thermoregulation, iatrogenic syndromes driven by polypharmacy, reduced mobility, and falls. In combination with osteoporosis, these lead to low-energy fractures. These limitations arise from progressive lifestyle-related diseases and many age-associated chronic conditions (17–19).

Population aging increases the incidence of time-critical events such as acute coronary syndromes, stroke, and severe low-energy trauma after falls. It also increases clinical complexity due to multimorbidity, frailty, and polypharmacy. Under these conditions, the air component of EMS is strategic. HEMS shortens time to scene in areas with limited ground access, provides advanced prehospital competencies including definitive airway management, ventilation, sedation, and rapid analgesia, and enables direct transfer to high-specialty centers while bypassing lower-level hospitals. These attributes are crucial in seniors, whose physiological reserve is limited and for whom delays more often lead to decompensation and iatrogenic complications.

In this context, HEMS dispatch sensitivity to mechanisms typical of older adults is essential. In older adluts, many severe events follow low-energy mechanisms such as a fall from less than 2 meters. Such patients often do not meet criteria for immediate HEMS dispatch yet ultimately require Prehospital Emergency Anaesthesia (PHEA) and transfer to a Major Trauma Center (MTC). This pattern suggests the need for dispatch triggers modulated by age and mechanism (20).

The state’s priority challenges and directions of support for seniors include development of assistive services, family-oriented information campaigns, institutional support for isolated older adults, urgent assistance and EMS, and HEMS interventions. These elements are analyzed in detail in this study. To date, no nationwide description of HEMS missions in patients aged 65 years and older has been published that simultaneously covers dispatch characteristics, diagnostic profiles, ALS pharmacotherapy and procedures, and seasonal and diurnal variation. It also remains unresolved whether age-related differences in escalation to PHEA and advanced airway management persist after accounting for case-mix, severity, and treatment limitation decisions- variables that are only partially captured in routine registries.

## Purpose

The objective was to provide a cross-sectional profile of HEMS interventions in Poland among patients aged ≥65 years between 2015 and 2024. The analysis covers diagnoses coded by the International Statistical Classification of Diseases and Related Health Problems, 10th Revision (ICD-10), dispatch reasons, Advanced Life Support (ALS)-level procedures, pharmacotherapy, severity scales, and destination of transport. Severity was assessed with the Glasgow Coma Scale (GCS), the Revised Trauma Score (RTS), and the National Advisory Committee for Aeronautics (NACA) score. We also examined operational patterns by time of day, day of the week, season, and urban versus rural setting, as well as age-related differences (65–74 vs. ≥ 75 years) in advanced interventions, with emphasis on advanced airway management and PHEA.

### Hypothesis

We hypothesized that patients aged ≥75 years, compared with those aged 65–74 years, would have lower rates of escalation to advanced airway management and to PHEA. Because frailty and baseline functional status were not measured, this hypothesis is framed as exploratory and hypothesis-generating rather than mechanistic.

## Materials and methods

### Study design

This was a 10-year retrospective analysis of HEMS missions. It was a multicenter observation covering all HEMS bases between 2015 and 2024, including permanent, seasonal, and newly opened bases. We included 30,075 HEMS missions, representing 31,9% of all missions completed nationwide. Data were drawn from HEMS medical and operational records. Missions were eligible if they met prespecified inclusion and exclusion criteria; specifically, we included completed dispatches with an identifiable patient aged ≥65 years and excluded records with missing key variables, unknown patients, and dispatches coded as dead on arrival (DOA), i.e., the patient was already dead when the crew reached the scene.

### Study setting

Poland operates 22 HEMS bases, both permanent and seasonal. Duty patterns include 24-h cover, 07:00–20:00, or sunrise-to-sunset. During the study period, four new permanent HEMS bases became operational in 2016: Ostrów Wielkopolski (2016), Gorzów Wielkopolski (2016), Sokołów Podlaski (2016), and Opole (2016). A HEMS crew consists of three members, reflecting a physician-staffed HEMS model: an EMS physician responsible for patient care, a paramedic or system nurse supporting medical care and performing ALS procedures, and a pilot operating the helicopter (21).

To enable international benchmarking, we calculated standardized HEMS provision metrics using the same denominators as European HEMS provision surveys (helicopters per million population and per 1,000 km^2^ land area). HEMS in Poland is delivered from 21 permanent bases and one seasonal base (22 in total) under the national system framework. For provision calculations, the base count was used as a proxy for the number of operationally deployed HEMS helicopters (one helicopter assigned per base), consistent with national operational descriptions. Following the European approach, provision was expressed relative to national population and land area. Using Statistics Poland (GUS) denominators (population 38.434 million; land area 311.9 thousand km^2^; administrative area 312.7 thousand km^2^), Poland’s provision equals 0.57 HEMS helicopters per million population and 0.07 per 1,000 km^2^ land area (approximately one base per 1.75 million residents and per 14,200 km^2^). These standardized metrics are therefore directly comparable to European surveys applying the same methodology. Operationally, an effective base operating radius of approximately 60 km (reported time-to-scene up to 20 min within this radius under typical conditions) provides additional context when interpreting land-area-based density metrics (22, 23).

### Variables

The prehospital diagnosis group variable classifies on-scene status into five clinical categories. Neurological emergencies include acute central nervous system events such as suspected stroke or TIA, seizures, sudden impairment of consciousness of neurological origin, and focal deficits. Cardiovascular emergencies include ACS, malignant arrhythmias, unstable angina, acute decompensated heart failure, and out-of-hospital cardiac arrest. Trauma includes blunt and penetrating injuries, low- and high-energy falls, and injuries of the head, chest, abdomen, spine, and limbs. Acute respiratory failure includes acute respiratory conditions with overt failure or high risk of failure, for example COPD or asthma exacerbations, severe pneumonia, cardiogenic pulmonary edema, and field-diagnosed tension pneumothorax. The “other” category covers internal medicine and metabolic conditions not meeting the above domains, as well as poisonings and environmental exposures when the care profile matches internal medicine interventions.

Dependent variables included prehospital diagnosis category, on-scene mortality after HEMS arrival (death on scene, coded 1/0), mission outcome mode (HEMS transport vs. EMS transport), and performance of ALS procedures coded as 0/1 indicators: manual chest compressions, mechanical compressions, sedation, neuromuscular blockade, electrotherapy (defibrillation, cardioversion, transcutaneous pacing), ultrasound, endotracheal intubation, supraglottic airway, oropharyngeal airway, mechanical ventilation, oxygen therapy, chest drainage, cervical collar, spinal board immobilization, IV/IO access, gastric tube, suction, and urinary catheterization. Composite variables were advanced airway management (endotracheal intubation or supraglottic airway or oropharyngeal airway) and PHEA (sedation with or without an induction agent with or without neuromuscular blockade). Medication groups were also analyzed as 0/1 indicators: fluids and electrolytes (0.9% NaCl, balanced crystalloids, Ringer’s solution, glucose), analgesics and antipyretics including opioids (metamizole, morphine, fentanyl), sympathomimetics and cardiotropics (adrenaline, noradrenaline, dopamine, dobutamine, ephedrine, atropine, furosemide, urapidil), antiemetics (ondansetron), sedatives and anticonvulsants (midazolam, diazepam), anesthetics and induction agents (etomidate, propofol, ketamine), neuromuscular blockers (for example rocuronium, suxamethonium), antiarrhythmics and antithrombotics (amiodarone, ASA, clopidogrel, heparin), hemostatic and metabolic agents (tranexamic acid, 8.4% sodium bicarbonate, dextrose boluses for hypoglycemia), and “other” agents (for example dexamethasone). We also analyzed severity scales GCS and RTS (continuous) and NACA 4–7 vs. 0–3 (binary), operational times and distances (continuous), ECG rhythm (categories: sinus rhythm, AF/AFl, asystole/PEA, SVT, bradycardia/AV block, VF/VT, other; with an additional asystole/PEA indicator 1/0), and clinical signs (cyanosis, dyspnea, apnea, meningeal signs, seizures, paresis; all 0/1).

Independent variables included age, sex, operational context variables, and mission type (HEMS primary vs. IFT). Mission type was treated primarily as an operational descriptor of case context; given fundamental differences in indications and workflow, we did not interpret ‘on-scene death after arrival’ as directly comparable between mission types. We defined the study population by an age threshold of ≥65 years. For comparative analyses we used two subgroups: 65–74 years and ≥75 years. This is a deliberate analytical convention widely used in clinical literature and health policy. It is not a formal WHO periodization.

**Figure 1:**
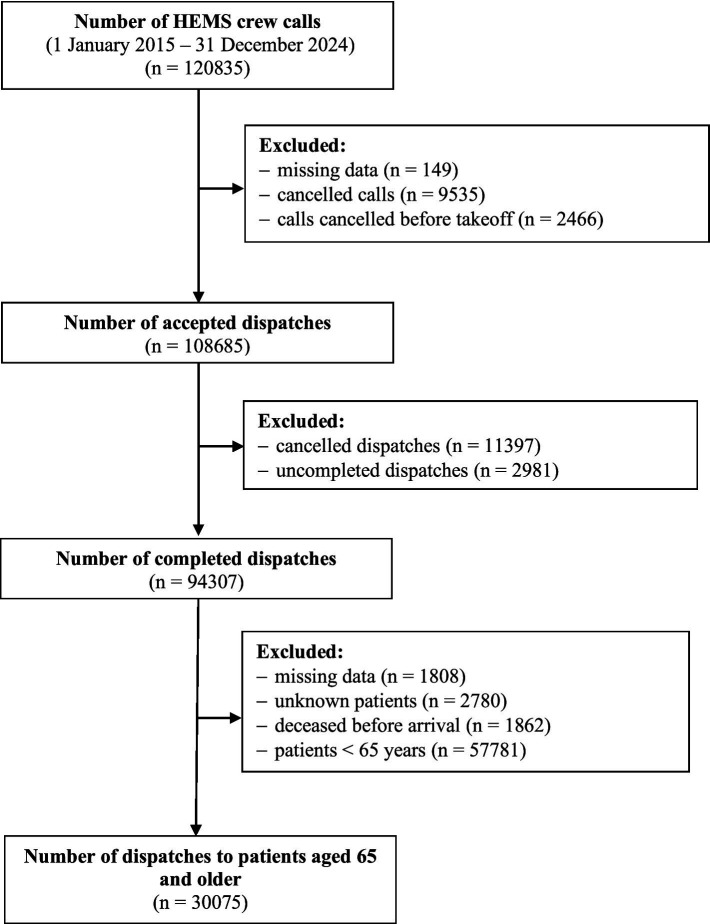
Study flow diagram: Selection of HEMS dispatches for patients aged ≥65 years.

Endpoint definition: In this study, prehospital mortality refers to on-scene death after HEMS arrival, operationalized as the registry-recorded mission outcome left on scene - death. Dispatches classified as DOA were excluded at cohort selection (Figure 1); therefore, mortality analyses pertain to patients who were alive when the crew reached the scene. This design necessarily conditions the mortality endpoint on survival until HEMS arrival and may introduce selection (survivor) bias if the probability of DOA differs by age, diagnosis, or dispatch pathway. The registry extract did not provide a separate, reliable indicator of death during transport or after hospital arrival; these events were not included in the endpoint.

Frailty was not directly measured in the registry (no standardized frailty scale or baseline functional status was available). Therefore, we did not operationalize a frailty or proxy frailty variable. Instead, age was analyzed in two strata (65–74 vs. ≥ 75 years) to explore heterogeneity of unadjusted associations, and neurological deficits (e.g., paresis) were treated as clinical presentation variables rather than markers of frailty. Analyses were performed at the level of individual components.

### Data collection

We prepared the database in Microsoft Excel using MS Office 2021 for Windows 11. We entered eligible missions with fields for mission type, date and time, mission duration, management, medical procedures, and pharmacotherapy. Data were obtained in electronic form as an Excel database. The stages of data acquisition and extraction are summarized in Figure 1.

### Statistical analysis

Statistical analyses were performed using IBM SPSS Statistics 25.0 for Windows (Armonk, NY: IBM Corp.). Categorical variables were reported as numbers (n) and percentages (%). Continuous variables were summarized as mean (SD) or median (IQR), depending on distribution and interpretability; in the tables we report mean (SD) for operational times and severity scales. The normality of distribution was assessed using the Kolmogorov–Smirnov test with the Lilliefors correction. Group comparisons were conducted using the χ^2^ test for categorical variables and the Mann–Whitney U test for continuous variables, or the Kruskal–Wallis test when more than two groups were analyzed. Associations between clinical–operational variables and age groups, as well as factors related to on-scene mortality after HEMS arrival, were assessed using univariable (unadjusted) logistic regression. Results were expressed as unadjusted odds ratios (OR) with 95% confidence intervals (95% CI). No multivariable (severity-adjusted) models were fitted; therefore, adjusted ORs (aOR) and multivariable model diagnostics are not applicable. Accordingly, all reported associations particularly those involving on-scene mortality should be interpreted as descriptive and potentially confounded by case-mix, measured and unmeasured illness severity, and treatment limitation decisions. Analyses were performed using complete-case data per model; no imputation was undertaken. A *p* < 0.05 was considered statistically significant.

### Ethical considerations

No data identifying patients, HEMS crews, or collaborating services were disclosed. All described cases are fully anonymized in accordance with the Declaration of Helsinki. The study received approval from the Director of the national HEMS operator and from the Bioethics Committee of the Medical University of Warsaw (AKBE/296/2025) on 13 October 2025.

## Results

During the study period, HEMS crews in Poland completed a total of 94,307 missions nationwide. Of these, 30,075 involved patients aged ≥65 years (mean age 75.7 ± 7.8 years), with a male predominance (55.9%). Most were HEMS primary missions (*n* = 25.901; 86.1%); IFT accounted for 13.9% (*n* = 4.174). Because these mission types represent fundamentally different operational contexts, we interpret pooled results as a system-level description rather than a direct comparison between mission types. On-scene death after HEMS arrival occurred almost exclusively in primary missions (1.964/25.901; 7.6%) and was rare in IFT (1/4,174; 0.02%); therefore, mortality findings are not interpreted as comparable across mission types. Dispatches were mainly during daytime (92.5%) and on weekdays (74.1%). Rural areas generated more calls (57.1%) than urban areas (42.9%). Most missions ended with HEMS transport to hospital (83.1%); EMS transport was less frequent (6.9%).

Neurological emergencies were the largest category (42.8%), followed by cardiovascular emergencies (31.0%), trauma (14.2%), acute respiratory disorders (3.7%), and other (8.3%). On ECG, atrial fibrillation/flutter was present in 16.3% and asystole/PEA in 6.4%. Severity scales indicated substantial clinical burden: mean GCS 10.1 ± 4.3, RTS 10.2 ± 3.6, and NACA 4.0 ± 1.7. Mean HEMS time-to-scene was 17.1 ± 6.4 min. The mean distance to scene was 45.4 ± 22.4 km; mean transport distance was 47.6 ± 27.2 km. Advanced procedures were frequent: endotracheal intubation 11.4%, mechanical ventilation 7.5%, oxygen therapy 12.0%, manual chest compressions 8.3%, mechanical compression 3.2%, and electrotherapy 2.3%. IV access was obtained in 35.6% and IO access in 0.9% of missions; see Table 1.

**Table 1:** Overall characteristics of the study population.

Parameter	Total (*n* = 30,075), *n* (%) or mean (SD)
Mission type– *n* (%)	
HEMS primary mission	25,901 (86.1)
Interfacility transfer	4,174 (13.9)
On-scene death after HEMS arrival – *n* (%)	
No	28,110 (93.5)
Yes	1965 (6.5)
Call time – *n* (%)	
07:00–18:59	27,827 (92.5)
19:00–06:59	2,248 (7.5)
Mission outcome– *n* (%)	
Transport to hospital by HEMS	24,994 (83.1)
Transport to hospital by EMS	2078 (6.9)
Left on scene – death	1965 (6.5)
Left on scene	1,038 (3.5)
Sex – *n* (%)	
Female	13,256 (44.1)
Male	16,819 (55.9)
Pre-hospital diagnostic groups – *n* (%)	
Neurological emergencies	12,880 (42.8)
Cardiovascular	9,332 (31.0)
Trauma	4,268 (14.2)
Respiratory emergencies	1,099 (3.7)
Other medical conditions	2,496 (8.3)
Year – *n* (%)	
2015	2,334 (7.8)
2016	2,228 (7.4)
2017	2,756 (9.2)
2018	3,180 (10.6)
2019	3,304 (11.0)
2020	3,194 (10.6)
2021	3,539 (11.8)
2022	3,150 (10.5)
2023	3,175 (10.6)
2024	3,215 (10.7)
Season– *n* (%)	
Spring	8,498 (28.3)
Summer	9,586 (31.9)
Autumn	6,985 (23.2)
Winter	5,006 (16.6)
Day type– *n* (%)	
Weekdays	22,282 (74.1)
Weekend	7,793 (25.9)
Call location – *n* (%)	
Rural	17,161 (57.1)
Urban	12,914 (42.9)
Medical procedures – *n* (%)	
Manual chest compressions	2,495 (8.3)
Mechanical chest compression device	968 (3.2)
Electrotherapy	682 (2.3)
Ultrasound	2,423 (8.1)
Endotracheal intubation	3,430 (11.4)
Supraglottic airway	164 (0.6)
Oropharyngeal airway insertion	675 (2.2)
Mechanical ventilation	2,252 (7.5)
Oxygen therapy	3,613 (12.0)
Chest drainage	193 (0.6)
Cervical collar	1,315 (4.4)
Spinal board immobilization	2,165 (7.2)
IV access	10,695 (35.6)
IO access	267 (0.9)
Gastric tube placement	279 (0.9)
Airway suction	1,373 (4.6)
Urinary catheter placement	556 (1.9)
ECG rhythm – *n* (%)	
Sinus rhythm	20,259 (67.4)
AF/AFl	4,899 (16.3)
Asystole/PEA	1916 (6.4)
SVT	1,082 (3.6)
Bradycardia/AV block	783 (2.6)
VF/VT	321 (1.1)
Other	815 (2.7)
Age – M (SD)	75.7 (7.8)
Age– *n* (%)	
65–74 years	15,179 (50.5)
≥ 75 years	14,896 (49.5)
Time to scene – M (SD)	17.1 (6.4)
Transport time – M (SD)	17.2 (7.7)
On-scene time – M (SD)	21.5 (13.0)
Total patient care time– M (SD)	47.4 (18.8)
Distance to scene – M (SD)	45.4 (22.4)
Transport distance – M (SD)	47.6 (27.2)
GCS – M (SD)	10.1 (4.3)
RTS – M (SD)	10.2 (3.6)
NACA – M (SD)	4.0 (1.7)
NACA – *n* (%)	
NACA 0–3 non-critical	7,375 (24.5)
NACA 4–7 critical emergency	22,700 (75.5)
Blood glucose level– M (SD)	161.3 (345.4)
Clinical signs – *n* (%)	
Cyanosis	1,592 (5.3)
Dyspnea	2,895 (9.6)
Apnea	3,485 (11.6)
Meningeal signs	324 (1.1)
Seizures	632 (2.1)
Paresis	9,019 (30.0)
Sedation – *n* (%)	4,797 (16.0)
Neuromuscular blockade– *n* (%)	2,308 (7.7)
Medications administered – *n* (%)	
Fluids and electrolytes	5,171 (17.2)
Analgesics and antipyretics	5,088 (16.9)
Sympathomimetic and cardiotropic agents	4,081 (13.6)
Antiemetics	3,656 (12.2)
Other	2,646 (8.8)
Sedatives and anticonvulsants	2,516 (8.4)
Anesthetic and induction agents	2,403 (8.0)
Neuromuscular blockers	2,175 (7.2)
Hemostatic and metabolic agents	1,593 (5.3)
Antiarrhythmics and antithrombotics	1,303 (4.3)

In patients aged ≥65 years, the five diagnostic groups showed distinct demographic, clinical, and operational profiles. In neurological cases, older age was more common (≥75 years: 57.6%) and women predominated (52.6%) (*p* < 0.001). Trauma peaked in summer (36.1%), whereas respiratory calls had a higher share in winter. Across diagnoses, most missions occurred on weekdays (74%) and during daytime (92%). Rural locations were more frequent in trauma (69.3%) and in the other category (63.1%) (*p* < 0.001).

Severity scales indicated the highest acuity in cardiovascular cases, with higher NACA (mean 4.5 ± 1.7; NACA 4–7 in 82.1%) and lower GCS and RTS (all *p* < 0.001). On-scene time was longest in respiratory cases (35.0 ± 21.3 min) and in trauma (24.7 ± 15.5 min). Transport distances were shortest in trauma (40.9 ± 23.2 km) and longest in the other category (61.3 ± 52.1 km) (*p* < 0.001).

By intervention profile, cardiovascular missions most often involved resuscitation (manual chest compressions 21.4%, mechanical compression 8.9%) and electrotherapy (6.3%). Trauma had high rates of intubation (19.2%), immobilization (cervical collar 28.2%; spine board 40.6%), and chest drainage (3.9%). In respiratory cases, oxygen therapy (26.8%) and mechanical ventilation (14.1%) predominated. Analgesics were most frequent in trauma (43.1%) (*p* < 0.001); see Table 2.

**Table 2:** Comparative analysis by diagnostic category.

Parameter	Neurological emergencies	Cardiovascular	Trauma	Respiratory emergencies	Other medical conditions	*p*-value
Age – *n* (%)						
65–74 years	5,465 (42.4)	5,192 (55.6)	2,752 (64.5)	527 (48.0)	1,243 (49.8)	<0.001
≥ 75 years	7,415 (57.6)	4,140 (44.4)	1,516 (35.5)	572 (52.0)	1,253 (50.2)	
Sex – *n* (%)						
Female	6,769 (52.6)	3,537 (37.9)	1,331 (31.2)	533 (48.5)	1,086 (43.5)	<0.001
Male	6,111 (47.4)	5,795 (62.1)	2,937 (68.8)	566 (51.5)	1,410 (56.5)	
Season – *n* (%)						
Spring	3,713 (28.8)	2,633 (28.2)	1,110 (26.0)	339 (30.8)	703 (28.2)	<0.001
Summer	4,055 (31.5)	2,943 (31.5)	1,542 (36.1)	267 (24.3)	779 (31.2)	
Autumn	2,874 (22.3)	2,195 (23.5)	1,084 (25.4)	260 (23.7)	572 (22.9)	
Winter	2,238 (17.4)	1,561 (16.7)	532 (12.5)	233 (21.2)	442 (17.7)	
Day type – *n* (%)						
Weekdays	9,455 (73.4)	6,946 (74.4)	3,228 (75.6)	802 (73.0)	1851 (74.2)	0.046
Weekend	3,425 (26.6)	2,386 (25.6)	1,040 (24.4)	297 (27.0)	645 (25.8)	
Call location – *n* (%)						
Rural	7,097 (55.1)	4,859 (52.1)	2,956 (69.3)	675 (61.4)	1,574 (63.1)	<0.001
Urban	5,783 (44.9)	4,473 (47.9)	1,312 (30.7)	424 (38.6)	922 (36.9)	
Call time – *n* (%)						
07:00–18:59	11,980 (93.0)	8,591 (92.1)	3,966 (92.9)	1,011 (92.0)	2,279 (91.3)	0.007
19:00–06:59	900 (7.0)	741 (7.9)	302 (7.1)	88 (8.0)	217 (8.7)	
Mission outcome – *n* (%)						
Transport to hospital by HEMS	11,987 (93.1)	7,052 (75.6)	3,622 (84.9)	690 (62.8)	1,643 (65.8)	<0.001
Transport to hospital by EMS	606 (4.7)	504 (5.4)	317 (7.4)	269 (24.5)	382 (15.3)	
Left on scene	275 (2.1)	186 (2.0)	63 (1.5)	123 (1.5)	391 (15.7)	
Left on scene – death	12 (0.1)	1,590 (17.0)	266 (6.2)	17 (1.5)	80 (3.2)	
NACA – M (SD)	3.9 (1.4)	4.5 (1.7)	3.9 (1.7)	3.8 (1.6)	3.5 (1.8)	<0.001
NACA – *n* (%)						
NACA 0–3 non-critical	2,983 (23.2)	1,671 (17.9)	1,319 (30.9)	352 (32.0)	1,050 (42.1)	<0.001
NACA 4–7 critical emergency	9,897 (76.8)	7,661 (82.1)	2,949 (69.1)	747 (68.0)	1,446 (57.9)	
GCS – M (SD)	12.4 (3.3)	11.4 (5.3)	12.2 (4.4)	12.6 (3.9)	12.6 (3.9)	<0.001
RTS – M (SD)	11.1 (1.8)	8.7 (5.0)	10.5 (3.2)	10.6 (2.8)	10.6 (3.0)	<0.001
Transport time – M (SD)	17.3 (6.3)	17.1 (7.4)	15.3 (6.6)	16.5 (11.3)	21.0 (14.6)	<0.001
On-scene time – M (SD)	18.2 (10.5)	22.7 (13.9)	24.7 (15.5)	35.0 (21.3)	28.9 (16.5)	<0.001
Total patient care time– M (SD)	43.8 (14.8)	49.9 (19.3)	48.1 (17.8)	57.3 (29.3)	54.2 (27.5)	<0.001
Transport distance – M (SD)	48.0 (22.0)	47.5 (26.0)	40.9 (23.2)	44.2 (39.0)	61.3 (52.1)	<0.001
Medical procedures – *n* (%)						
Manual chest compressions	36 (0.3)	1996 (21.4)	337 (7.9)	31 (2.8)	95 (3.8)	<0.001
Mechanical chest compression device	7 (0.1)	830 (8.9)	82 (1.9)	11 (1.0)	38 (1.5)	<0.001
Ultrasound	140 (1.1)	1,021 (10.9)	1,016 (23.8)	85 (7.7)	161 (6.5)	<0.001
Endotracheal intubation	763 (5.9)	1,485 (15.9)	819 (19.2)	107 (9.7)	256 (10.3)	<0.001
Supraglottic airway	11 (0.1)	120 (1.3)	24 (0.6)	2 (0.2)	7 (0.3)	<0.001
Oropharyngeal airway insertion	202 (1.6)	269 (2.9)	138 (3.2)	19 (1.7)	47 (1.9)	<0.001
Mechanical ventilation	489 (3.8)	842 (9.0)	524 (12.3)	155 (14.1)	242 (9.7)	<0.001
Oxygen therapy	1,105 (8.6)	1,235 (13.2)	680 (15.9)	295 (26.8)	298 (11.9)	<0.001
Chest drainage	3 (0.0)	15 (0.2)	167 (3.9)	5 (0.5)	3 (0.1)	<0.001
Cervical collar	41 (0.3)	29 (0.3)	1,205 (28.2)	3 (0.3)	37 (1.5)	<0.001
Spinal board immobilization	163 (1.3)	157 (1.7)	1734 (40.6)	14 (1.3)	97 (3.9)	<0.001
IV access	3,979 (30.9)	3,285 (35.2)	1735 (40.7)	635 (57.8)	1,061 (42.5)	<0.001
IO access	8 (0.1)	151 (1.6)	73 (1.7)	6 (0.5)	29 (1.2)	<0.001
Gastric tube placement	36 (0.3)	139 (1.5)	38 (0.9)	16 (1.5)	50 (2.0)	<0.001
Airway suction	299 (2.3)	571 (6.1)	313 (7.3)	87 (7.9)	103 (4.1)	<0.001
Urinary catheter placement	185 (1.4)	167 (1.8)	69 (1.6)	44 (4.0)	91 (3.6)	<0.001
Sedation	1,610 (12.5)	1,202 (12.9)	1,176 (27.6)	284 (25.8)	525 (21.0)	<0.001
Neuromuscular blockade	708 (5.5)	490 (5.3)	688 (16.1)	140 (12.7)	282 (11.3)	<0.001
Electrotherapy	17 (0.1)	589 (6.3)	52 (1.2)	3 (0.3)	21 (0.8)	<0.001
Medications administered – *n* (%)						
Fluids and electrolytes	1,474 (11.4)	1,600 (17.1)	1,034 (24.2)	208 (18.9)	855 (34.3)	<0.001
Analgesics and antipyretics	847 (6.6)	1,587 (17.0)	1839 (43.1)	246 (22.4)	569 (22.8)	<0.001
Sympathomimetic and cardiotropic agents	615 (4.8)	2,311 (24.8)	568 (13.3)	329 (29.9)	258 (10.3)	<0.001
Antiemetics	2080 (16.1)	879 (9.4)	476 (11.2)	45 (4.1)	176 (7.1)	<0.001
Other	702 (5.5)	1,181 (12.7)	262 (6.1)	267 (24.3)	234 (9.4)	<0.001
Sedatives and anticonvulsants	975 (7.6)	673 (7.2)	501 (11.7)	133 (12.1)	234 (9.4)	<0.001
Anesthetic and induction agents	752 (5.8)	443 (4.7)	804 (18.8)	127 (11.6)	277 (11.1)	<0.001
Neuromuscular blockers	668 (5.2)	472 (5.1)	685 (16.0)	113 (10.3)	237 (9.5)	<0.001
Hemostatic and metabolic agents	135 (1.0)	611 (6.5)	613 (14.4)	125 (11.4)	109 (4.4)	<0.001
Antiarrhythmics and antithrombotics	44 (0.3)	1,194 (12.8)	18 (0.4)	24 (2.2)	23 (0.9)	<0.001

Statistical analysis showed significant differences by age. In patients aged ≥75 years, the odds of neurological missions were higher (OR 1.34; 95% CI 1.24–1.47; *p* < 0.001). The odds were lower for cardiovascular missions (OR 0.79; 95% CI 0.72–0.86; *p* < 0.001) and for trauma (OR 0.54; 95% CI 0.49–0.60; *p* < 0.001). Compared with patients aged 65–74 years, those ≥75 years had 25% lower odds of on-scene death after HEMS arrival (OR 0.75; 95% CI 0.69–0.83; *p* < 0.001). This association is counterintuitive and should be interpreted cautiously because it is unadjusted and may be driven by differences in case-mix, dispatch/selection processes, and the endpoint definition (including exclusion of DOA and lack of capture of deaths during transport or after hospital arrival).

In analyses stratified by age (65–74 vs. ≥ 75 years), patients ≥75 years less often received components of escalated anesthetic–airway care: intubation (OR 0.71; 95% CI 0.66–0.76), sedation (OR 0.76; 95% CI 0.71–0.80), and neuromuscular blockade (OR 0.66; 95% CI 0.61–0.72), all *p* < 0.001. The direction was consistent for mechanical ventilation (OR 0.60; 95% CI 0.55–0.65) and oxygen therapy (OR 0.80; 95% CI 0.74–0.86). All reported ORs are unadjusted (univariable) associations and may be influenced by differences in case-mix and illness severity.

For procedures and advanced interventions, patients ≥75 years less often underwent manual chest compressions (OR 0.70; 95% CI 0.64–0.76; *p* < 0.001), mechanical compression (OR 0.64; 95% CI 0.56–0.73; *p* < 0.001), electrotherapy (OR 0.69; 95% CI 0.59–0.80; *p* < 0.001), intubation (OR 0.71; 95% CI 0.66–0.76; *p* < 0.001), mechanical ventilation (OR 0.60; 95% CI 0.55–0.65; *p* < 0.001), and oxygen therapy (OR 0.80; 95% CI 0.74–0.86; *p* < 0.001); see Table 3.

**Table 3:** Univariable logistic regression (unadjusted OR) of clinical–operational variables by patient age.

Parameter	65–74 years	≥ 75 years	p-value	Unadjusted OR (95% CI)
Pre-hospital diagnostic groups – *n* (%)				
Neurological emergencies	5,465 (36.00)	7,415 (49.78)	<0.001	1.34*** (1.24–1.47)
Cardiovascular	5,192 (34.22)	4,140 (27.81)		0.79*** (0.72–0.86)
Trauma	2,752 (18.13)	1,516 (10.18)		0.54*** (0.49–0.60)
Respiratory emergencies	527 (3.52)	572 (3.84)		1.08 ^ns^ (0.93–1.24)
Other medical conditions	1,243 (8.23)	1,253(8.44)		Ref.
Mission outcome – *n* (%)				
Transport to hospital by HEMS	12,646 (83.3)	12,348 (82.9)	<0.001	Ref.
Transport to hospital by EMS	1,007 (6.6)	1,071 (7.2)		1.09 ^ns^ (0.99–1.19)
Left on scene – death	1,121 (7.4)	844 (5.7)		0.77*** (0.70–0.85)
Left on scene	405 (2.7)	633 (4.2)		1.60*** (1.41–1.82)
On-scene death after HEMS arrival– *n* (%)				
No	14,058 (92.6)	14,052 (94.3)	<0.001	Ref.
Yes	1,121 (7.4)	844 (5.7)		0.75 (0.69–0.83)
Sex – *n* (%)				
Female	5,017 (33.1)	8,239 (55.3)	<0.001	Ref.
Male	10,162 (66.9)	6,657 (44.7)		0.40 (0.38–0.42)
Call location– *n* (%)				
Rural	8,521 (56.1)	8,640 (58.0)	0.001	Ref.
Urban	6,658 (43.9)	6,256 (42.0)		0.93 (0.89–0.97)
Day type– *n* (%)				
Weekdays	11,354 (74.8)	10,928 (73.4)	0.004	Ref.
Weekend	3,825 (25.2)	3,968 (26.6)		1.08 (1.02–1.14)
Medical procedures– *n* (%)				
Manual chest compressions	1,462 (9.6)	1,033 (6.9)	<0.001	0.70 (0.64–0.76)
Mechanical chest compression device	592 (3.9)	376 (2.5)	<0.001	0.64 (0.56–0.73)
Electrotherapy	406 (2.7)	276 (1.9)	<0.001	0.69 (0.59–0.80)
Ultrasound	1,514 (10.0)	909 (6.1)	<0.001	0.59 (0.54–0.64)
Endotracheal intubation	1993 (13.1)	1,437 (9.6)	<0.001	0.71 (0.66–0.76)
Supraglottic airway	109 (0.7)	55 (0.4)	<0.001	0.51 (0.37–0.71)
Oropharyngeal airway insertion	366 (2.4)	309 (2.1)	0.049	0.86 (0.74–1.00)
Mechanical ventilation	1,401 (9.2)	851 (5.7)	<0.001	0.60 (0.55–0.65)
Oxygen therapy	2003 (13.2)	1,610 (10.8)	<0.001	0.80 (0.74–0.86)
Chest drainage	121 (0.8)	72 (0.5)	0.001	0.60 (0.45–0.81)
Cervical collar	863 (5.7)	452 (3.0)	<0.001	0.52 (0.46–0.58)
Spinal board immobilization	1,396 (9.2)	769 (5.2)	<0.001	0.54 (0.49–0.59)
IV access	5,273 (34.7)	5,422 (36.4)	0.003	1.08 (1.03–1.13)
IO access	170 (1.1)	97 (0.7)		0.58 (0.45–0.74)
Gastric tube placement	180 (1.2)	99 (0.7)	<0.001	0.56 (0.44–0.71)
Airway suction	783 (5.2)	590 (4.0)	<0.001	0.76 (0.68–0.85)
Urinary catheter placement	294 (1.9)	262 (1.8)	0.252	0.91 (0.77–1.07)
NACA – M (SD)	4.1 (1.7)	4.0 (1.6)	<0.001	-
NACA – *n* (%)				
NACA 0–3 non-critical	3,674 (24.2)	3,701 (24.8)	0.196	Ref.
NACA 4–7 critical emergency	11,505 (75.8)	11,195 (75.2)		0.97 (0.92–1.02)
Clinical signs – *n* (%)				
Cyanosis	926 (6.1)	666 (4.5)	<0.001	0.72 (0.65–0.80)
Dyspnea	1,476 (9.7)	1,419 (9.5)	0.561	0.98 (0.91–1.06)
Apnea	1966 (13.0)	1,519 (10.2)	<0.001	0.76 (0.71–0.82)
Meningeal signs	151 (1.0)	173 (1.2)	0.162	1.17 (0.94–1.46)
Seizures	350 (2.3)	282 (1.9)	0.013	0.82 (0.70–0.96)
Paresis	3,863 (25.5)	5,156 (34.6)	<0.001	1.55 (1.48–1.63)
Sedation – n (%)	2,703 (17.8)	2094 (14.1)	<0.001	0.76 (0.71–0.80)
Neuromuscular blockade – *n* (%)	1,382 (9.1)	926 (6.2)	<0.001	0.66 (0.61–0.72)
Medications administered – *n* (%)				
Fluids and electrolytes	2,686 (17.7)	2,485 (16.7)	0.020	0.93 (0.88–0.99)
Analgesics and antipyretics	3,086 (20.3)	2002 (13.4)	<0.001	0.61 (0.57–0.65)
Sympathomimetic and cardiotropic agents	2,277 (15.0)	1804 (12.1)	<0.001	0.78 (0.73–0.84)
Antiemetics	1768 (11.7)	1888 (12.7)	0.006	1.10 (1.03–1.18)
Other	1,442 (9.5)	1,204 (8.1)	<0.001	0.84 (0.77–0.91)
Sedatives and anticonvulsants	1,385 (9.1)	1,131 (7.6)	<0.001	0.82 (0.76–0.89)
Anesthetic and induction agents	1,426 (9.4)	977 (6.6)	<0.001	0.68 (0.62–0.74)
Neuromuscular blockers	1,300 (8.6)	875 (5.9)	<0.001	0.67 (0.61–0.73)
Hemostatic and metabolic agents	932 (6.1)	661 (4.4)	<0.001	0.71 (0.64–0.79)
Antiarrhythmics and antithrombotics	824 (5.4)	479 (3.2)	<0.001	0.58 (0.52–0.65)
GCS – M (SD)	12.1 (4.4)	12.1 (4.1)	<0.001	–
RTS – M (SD)	10.1 (3.8)	10.4 (3.4)	0.003	–
Blood glucose level– M (SD)	166.6 (494.8)	156.5 (66.4)	0.028	–

*p < 0.05; **p < 0.01; ***p < 0.001; ns - not significant.

Cardiovascular diagnoses were associated with sixfold higher odds of death (OR 6.20; 95% CI 4.93–7.80; *p* < 0.001). Trauma was associated with nearly twofold higher odds (OR 2.01; 95% CI 1.56–2.59; *p* < 0.001).

Severity scales showed very large and statistically significant differences. NACA 4–7 versus 0–3 was linked to more than threefold higher odds of death (OR 3.27; 95% CI 2.80–3.81; *p* < 0.001). GCS values (12.6 ± 3.7 vs. 3.2 ± 1.4, *p* < 0.001) and RTS values (10.9 ± 2.6 vs. 0.5 ± 2.0, *p* < 0.001) clearly indicate extreme severity among patients who died; see Table 4.

**Table 4:** Univariable logistic regression (unadjusted OR) of clinical–operational variables versus on-scene mortality after HEMS arrival.

Parameter	Survived	Did not survive	p-value	Unadjusted OR (95% CI)
Pre-hospital diagnostic groups – *n* (%)				
Neurological emergencies	12,868 (45.8)	12 (0.6)	<0.001	0.03*** (0.02–0.05)
Cardiovascular	7,742 (27.5)	1,590 (80.9)		6.20*** (4.93–7.80)
Trauma	4,002 (14.2)	266 (13.5)		2.01*** (1.56–2.59)
Respiratory emergencies	1,082(3.8)	17 (0.9)		0.47** (0.28–0.81)
Other medical conditions	2,416 (8.6)	80 (4.1)		Ref.
Sex – *n* (%)				
Male	15,445 (54.9)	1,374 (69.9)	<0.001	Ref.
Female	12,665 (45.1)	591 (30.1)		0.53 (0.48–0.58)
Age – M (SD)	75.7 (7.8)	74.5 (7.4)	<0.001	-
Age – *n* (%)				
65–74 years	14,058 (50.0)	1,121 (57.0)	<0.001	Ref.
≥ 75 years	14,052 (50.0)	844 (43.0)		0.75 (0.69–0.83)
Call location – *n* (%)				
Rural	15,748 (56.0)	1,413 (71.9)	<0.001	Ref.
Urban	12,362 (44.0)	552 (28.1)		0.50 (0.45–0.55)
Day type– *n* (%)				
Weekdays	20,860 (74.2)	1,422 (72.4)	0.072	Ref.
Weekend	7,250 (25.8)	543 (27.6)		0.91 (0.82–1.01)
NACA – M (SD)	3.9 (1.5)	6.2 (1.8)	<0.001	-
NACA – *n* (%)				
NACA 0–3 non-critical	7,188 (25.6)	187 (9.5)	<0.001	Ref.
NACA 4–7 critical emergency	20,922 (74.4)	1778 (90.5)		3.27 (2.80–3.81)
Medications administered – *n* (%)				
Fluids and electrolytes	4,568 (16.3)	603 (30.7)	<0.001	2.28 (2.06–2.52)
Analgesics and antipyretics	5,045 (18.0)	43 (2.2)	<0.001	0.10 (0.08–0.14)
Sympathomimetic and cardiotropic agents	2,732 (9.7)	1,349 (68.7)	<0.001	20.35 (18.35–22.55)
Antiemetics	3,653 (13.0)	3 (0.2)	<0.001	0.01 (0.00–0.03)
Other	2,366 (8.4)	280 (14.3)	<0.001	1.81 (1.58–2.07)
Sedatives and anticonvulsants	2,495 (8.9)	21 (1.1)	<0.001	0.11 (0.07–0.17)
Anesthetic and induction agents	2,354 (8.4)	49 (2.5)	<0.001	0.28 (0.21–0.37)
Neuromuscular blockers	2,135 (7.6)	40 (2.0)	<0.001	0.25 (0.18–0.35)
Hemostatic and metabolic agents	1,224 (4.4)	369 (18.8)	<0.001	5.08 (4.47–5.77)
Antiarrhythmics and antithrombotics	1,053 (3.7)	250 (12.7)	<0.001	3.75 (3.24–4.34)
GCS – M (SD)	12.6 (3.7)	3.2 (1.4)	<0.001	–
RTS – M (SD)	10.9 (2.6)	0.5 (2.0)	<0.001	–
Blood glucose level – M (SD)	159.4 (354.9)	193.2 (98.6)	<0.001	–

*p < 0.05; **p < 0.01; ***p < 0.001; ns - not significant. The mortality endpoint reflects death on scene after crew arrival (mission outcome ‘left on scene – death’). Dispatches coded as DOA were excluded (Figure 1). Death during transport or after hospital arrival was not captured in the registry extract and is not included. Mission type was not modeled for mortality because ‘on-scene death after arrival’ is not operationally comparable between primary missions and IFT and because events were extremely sparse in IFT.

## Discussion

HEMS crews together with ground EMS teams form the interventional arm of the EMS system. The remaining functional units are stationary, including EDs and Major Trauma Centers. Mobile units deliver prehospital interventions. Ground EMS operates locally, while HEMS covers both local and long-range missions using the air route. Missions include responses to acute illness and accidents and interfacility transport. HEMS also transports healthcare staff, blood, other medical devices and medicinal products, and transplant consignments. Our study did not include this segment of HEMS activity, as stated in the Limitations (4). In our data, HEMS performed 30,075 of missions for seniors (31,9% of all missions), with activity concentrated during daytime (92.5%) and on weekdays (74.1%). This operational profile reflects ground accessibility and base duty hours. It indicates a high daytime workload for the air component, which matters for duty planning and maintaining personnel and equipment readiness.

In European comparative provision surveys, HEMS system capacity is commonly operationalized as the number of primary HEMS helicopters per million population and per 1,000 km^2^ land area (and, in some analyses, per GDP), with separate reporting of daytime and night-time capability where available. In the 2016 Europe-wide survey by Jones et al. (24), Poland was reported to have a maximum of 22 primary HEMS helicopters in service (reflecting seasonal variation), corresponding to 0.5698 helicopters per million population and 0.0704 per 1,000 km^2^. Across all surveyed countries, provision varied widely (including countries with no dedicated HEMS at all), with mean values of 0.85 helicopters per million population and 0.090 per 1,000 km^2^. These denominators therefore place Poland in a mid-range European context. However, provision densities are first-order descriptors only and cannot be used to infer an ‘optimal’ national HEMS capacity; required provision depends on the geographic distribution of incidents, the location of receiving hospitals, and the clinical/logistical criteria for HEMS activation, with additional constraints related to night-flying capability and other operational limitations. Jones et al. further note that night-flying capability is not universal across Europe; Poland is listed among countries with at least some aircraft operational around the clock, but the authors highlight incomplete data on night capability for some or all aircraft in several countries, including Poland. This is relevant for interpreting our predominantly daytime workload (92.5% of missions) and for future benchmarking, where explicit day-versus-night provision reporting would improve comparability.

Beyond aircraft density metrics, cross-country comparisons of HEMS performance and intervention profiles should explicitly consider clinical staffing models. In the HEMS Medical Crew Survey (67 eligible service submissions across Europe and other regions), there was no single standard: 73.1% of responding services reported physician involvement in the medical crew, and the three most common two-clinician models were physician plus HEMS crew member (26.9%), physician plus nurse (23.9%), and nurse plus EMT/paramedic (16.4%). The authors caution that response rates differed between countries; therefore, the survey is not necessarily fully representative at national level, but it provides a structured impression of the diversity of crew concepts (25).

Within this heterogeneous European landscape, Poland’s physician-staffed three-person configuration (physician + paramedic/system nurse + pilot) aligns with physician-involved HEMS traditions observed in several EU systems. For example, German operators describe a standard crew of pilot, emergency physician (Notarzt) and a clinical crew member (Rettungsfachpersonal/Notfallsanitäter), with a hoist operator added for selected mission profiles; similarly, the Austrian ÖAMTC (Christophorus) air rescue service describes a three-person crew consisting of flight rescue physician, flight rescuer and pilot. In contrast, the North American literature has historically described most helicopter EMS programs as deploying non-physician clinical crews, with a nurse/paramedic mix commonly reported as the predominant team, while physician-staffed programs exist but represent a smaller subset. These structural differences are a plausible contributor to between-system variation in dispatch thresholds and the practical availability of advanced interventions (e.g., PHEA and advanced airway management) and should be considered when benchmarking intervention rates internationally (26–28).

We combined two themes: HEMS interventions and the 65 + population, in line with the widely observed trend of population aging. Many authors have noted that aging is global. The trend is faster in high-income countries, including Poland and other European Union states, driven by longer life expectancy for both sexes, better medical care, improved diagnostics, more effective therapies and drug generations, prevention, lifestyle and diet change, higher hygiene, and vaccination (29, 30). In our ≥65 cohort, neurological emergencies dominated (42.8%), followed by cardiovascular emergencies (31.0%). This aligns with the burden profile of aging societies. The age bands 65–74 and ≥75 years were almost equally represented (50.5% vs. 49.5%). There was a female predominance in neurological cases (52.6%) and a male predominance in cardiovascular cases (62.1%). These patterns may reflect sex differences in risk exposures and disease biology. More than half of missions in our ≥65 cohort occurred in rural areas (57.1%), and trauma missions were predominantly rural (69.3%). This pattern is consistent with but does not prove the interpretation that HEMS is preferentially utilized where geography and access constraints reduce the effectiveness of ground-only pathways. Two non-mutually exclusive mechanisms merit consideration. First, rural dispatches may reflect longer prehospital distances and time components to definitive care in rural settings. Supporting this interpretation, a nationwide Polish comparative analysis of rural versus urban HEMS missions reported significantly longer flight-to-scene, on-scene and transport times, as well as longer distances to the scene and to the receiving hospital in rural missions, suggesting that geography contributes to dispatch patterns in Poland. Second, the trauma–rural skew may also reflect differences in injury epidemiology and operational feasibility: high-energy mechanisms on rural roads, agricultural/forestry exposures, delayed discovery, and more available landing sites may increase the likelihood of HEMS activation for trauma in rural locations. Notably, in our dataset trauma had the shortest mean transport distance among diagnostic groups, indicating that distance alone is unlikely to fully explain the trauma–rural association and that dispatch policy and case-mix differences may also contribute (31, 32).

In this nationwide cohort restricted to patients aged ≥65 years (including both primary missions and interfacility transfers), neurological (42.8%) and cardiovascular (31.0%) indications predominated, while trauma remained a substantial minority (14.2%). This case-mix has practical implications for dispatch logic, destination triage, and the competency framework required of HEMS crews caring for older adults. First, it suggests that dispatch and destination-triage logic in older adults should be optimized primarily around time-critical neurovascular and cardiac pathways (e.g., suspected stroke/TIA, seizures with persistent altered mental status, acute coronary syndromes, malignant arrhythmias, and out-of-hospital cardiac arrest), while retaining robust trauma triage for both high-energy and geriatric low-energy mechanisms. Second, it reinforces the need for clear, protocolised bypass and pre-notification interfaces with specialist receiving networks (stroke centers, catheterisation laboratories, and major trauma centers), because for a large share of missions the clinical value proposition of HEMS is not only ‘faster transport’ but also early advanced stabilization plus accurate destination matching. From a competency perspective, this case-mix implies that training, governance, and continuous skills maintenance should prioritize: (1) neuro-emergency assessment and decision support (structured neurological examination, recognition of stroke mimics, seizure management, airway protection strategies in reduced GCS, and safe procedural sedation); (2) advanced cardiovascular and resuscitation competencies (12-lead ECG interpretation, management of brady−/tachyarrhythmias including pacing/cardioversion/defibrillation, high-quality CPR logistics, and post-ROSC stabilization); and (3) trauma-critical care skills tailored to older adults (analgesia, hemorrhage control adjuncts, chest injury recognition and decompression, immobilization when indicated, and integration with major trauma triage). The observed procedural profile in our cohort (e.g., intubation, ventilation, resuscitative interventions, ultrasound) supports that these competencies are not ‘rare events’ and therefore justify formalized training pathways and quality assurance focusing on high-risk, low-frequency procedures (notably advanced airway management and PHEA components).

Finally, the above system-design considerations align with ongoing European efforts to improve comparability and quality across HEMS and air ambulance services. The European HEMS & Air Ambulance Committee (EHAC) positions itself as an umbrella organization engaging with regulators (including EASA) and promoting optimization of crew training and the development of uniform quality standards for equipment and operations. In parallel, EHAC’s Medical Working Group has initiated a peer-reviewed series of ‘best practice advice’ papers (e.g., on pre-hospital emergency anaesthesia and major incident critical care), explicitly aiming to support a common understanding of best practice across European services. In this context, reporting standardized provision metrics and transparently describing staffing and competency frameworks provides not only local system insight but also inputs for European benchmarking and harmonization (33, 34).

Population aging is tracked not only by researchers but also by statistical agencies. Eurostat reports a growing share of older adults with forecasts of further increases (7). Our study also showed a slight upward trend in annual mission counts.

Linder et al. studied geriatric patients in EDs. The most common conditions were cardiovascular disease, trauma, poisoning, and consciousness disorders, followed by gastrointestinal and respiratory disease and neurological disorders. Our observations and Table 4 confirm these threats to health (35, 36). We add that in seniors, cardiovascular missions carried sixfold higher odds of prehospital death (OR 6.20; 95% CI 4.93–7.80), while trauma carried nearly twofold higher odds (OR 2.01; 95% CI 1.56–2.59). These findings extend ED data into the prehospital domain. In the 65 + population, a cardiovascular phenotype appears to be the main driver of field mortality. This should prioritize rapid diagnostic pathways and time-critical interventions. Age ≥75 years was associated with less frequent escalation to advanced procedures, with lower ORs for mechanical compressions, intubation, ventilation, and oxygen therapy, along with lower odds of on-scene death after HEMS arrival versus the 65–74 group (OR 0.75). This counterintuitive finding requires cautious interpretation. First, the endpoint conditions on survival until HEMS arrival and excludes DOA dispatches; if DOA frequency differs by age or diagnosis, selection (survivor) bias may distort the apparent age–mortality association. Second, case-mix differs between age strata (e.g., differing proportions of cardiovascular versus neurological missions), and cardiovascular presentations carried the highest on-scene mortality in our dataset. Third, unmeasured factors comorbidity burden, pre-existing care limitations (DNR/palliative status), and dispatch/triage decisions may contribute. Because analyses are unadjusted, we cannot infer an independent protective effect of older age on mortality.

Frailty and baseline functional vulnerability may be relevant but were not measured in this registry. Prehospital literature supports this view. Conceptual and systematic reviews emphasize that decisions in older adults should consider social, functional, and pharmacologic factors rather than only the acute diagnosis. Ignoring these factors worsens outcomes, including higher ED return rates. Geriatric-oriented models therefore stress early risk stratification and tailored interventions for older adults, particularly when baseline vulnerability and functional needs are relevant (37).

Although our registry did not include formal frailty assessment, a HEMS feasibility study from the UK suggests that the Clinical Frailty Scale (CFS) can be recorded routinely in many patients ≥65 years, including those with low GCS. Notably, PHEA was not performed in patients with CFS ≥ 6, and direct transport to high-specialty centers occurred only in isolated cases. In that cohort, this pattern may reflect clinical caution in patients with higher frailty scores. The authors call for validation and for evaluation of how CFS shapes decisions and outcomes. Given rising HEMS volumes in seniors, we propose piloting CFS and evaluating it in Poland (6). Prior literature suggests that structured geriatric measures (including frailty instruments) may be prognostic and could potentially relate to escalation decisions in prehospital care; however, these constructs were not captured in our registry and cannot be evaluated here. In older adults with minor head injury and GCS 15, ground EMS transported about 70.5% of patients ≥65 years to EDs, with 81% meeting formal criteria under NICE/JRCALC. Decisions in the remainder were shaped by social context, lack of alternative care pathways, and uncertainty about pharmacotherapy. In an audit, CFS was recorded in 78 of 99 cases, and CFS ≥ 7 in about 9%. The authors suggest that for such frail patients, restorative or palliative strategies may be more appropriate than default escalation. These external data support the feasibility of capturing CFS in prehospital settings and motivate future registry enhancements to allow formal evaluation of whether baseline vulnerability and standardized geriatric measures are associated with escalation decisions and outcomes in HEMS (38).

Operationally, our observations fit the issue of under-sensitive classic dispatch criteria in older adults. Our Polish data, with heavy daytime activity, rural predominance, and high ALS intensity, align with AAKSS findings (39). In patients ≥65 years, PHEA was performed more often after crew-request dispatch (26.6%) than after immediate dispatch. Of these, 69.7% were taken directly to a Major Trauma Center. This indicates that some seniors who do not meet immediate-dispatch criteria still need advanced prehospital teams. Age-modified triggers, for example a fall <2 m with age ≥75 years and neurological symptoms, could improve dispatch sensitivity without inappropriate overtriage.

The apparent discrepancy may reflect phenotype selection, with more neurology and fewer acute cardiac cases, and a more conservative procedural strategy in older patients. While these descriptive findings are consistent with the potential value of improved geriatric data capture (e.g., baseline functional status and, where feasible, structured instruments such as CFS) in HEMS documentation, frailty was not measured directly and the present analyses are unadjusted; therefore, inferences about frailty-driven decision-making should remain cautious. In the ≥75 group, we observed lower odds of escalation of anesthetic and airway interventions, including intubation, sedation, and neuromuscular blockade (all OR<1; *p* < 0.001). These are age-stratified, unadjusted associations and should not be interpreted as evidence of frailty-driven decision-making. Convergence across multiple components suggests a consistent pattern, but causality cannot be inferred; residual confounding (e.g., case-mix, illness severity, and treatment limitations) may contribute. A missing variable on the type of destination center prevents testing the second part of the original hypothesis, that is direct transport to high-specialty centers. This remains a key direction for registry expansion.

Consistent with Table 2, cardiovascular cases showed higher NACA, lower GCS and RTS, and greater use of resuscitative and electrical interventions. Asystole/PEA was a strong predictor of poor outcome, translating into sixfold higher odds of death.

Martinelli discusses polypharmacy in seniors combined with physical and psychological burdens that hinder independent function. Key deficits include dementias, late-life depression, urinary and fecal incontinence, impaired hearing and vision, disturbed thermoregulation, iatrogenic syndromes, and low-energy trauma on the background of osteoporosis. Pharmacotherapy was analyzed in our study. Prehospital agents are acute-use medications. Seniors take daily chronic medications prescribed by primary care or specialists. Our data justify secondary analyses to assess polypharmacy risk in relation to chronic vs. emergency pharmacotherapy (40–42). Prehospital pharmacotherapy showed high use of sympathomimetic and cardiotropic agents in critical situations, with significant overrepresentation among deaths. In this subgroup there was less use of analgesics and sedatives, reflecting prioritization of cardio-respiratory stabilization over symptomatic relief. Trauma showed a medication profile with more frequent analgesia, consistent with the need for pain control and immobilization.

Trauma is a major threat in geriatric patients. Alshibani (43) reported that both the median age of trauma patients and the share with major trauma aged ≥65 years have increased in high-income countries. About 3% of older trauma patients were transported to trauma centers by HEMS when distance criteria were met, that is ≥60 min from the nearest trauma center. Our study included transport time, distance, and the clinical criterion of trauma. We found the shortest transport distances in trauma (40.9 ± 23.2 km), with trauma accounting for 14.2% of the study population. Trauma calls were more often rural (69.3%). On-scene times in trauma were 24.7 ± 15.5 min, and transport distances were the shortest among categories. The interplay between time and distance in trauma may reflect the high death risk in patients ≥65 years with trauma (OR 2.01; 95% CI 1.56–2.59; *p* < 0.001). Severity scales and ECG rhythms strongly differentiated death risk. NACA 4–7 versus 0–3 tripled the odds of death (OR 3.27). Asystole/PEA was a powerful marker of poor outcome. Cardiovascular cases had higher NACA, lower GCS and RTS, and the greatest intensity of resuscitative and electrical interventions (manual chest compressions 21.4%, mechanical compression 8.9%, electrotherapy 6.3%). This confirms that in geriatric prehospital care the highest death risk usually arises from a cardiovascular phenotype.

To further contextualize the trauma segment of our cohort, future comparative work could leverage the German TraumaRegister DGU® (TR-DGU), a national quality-assurance registry. TR-DGU documents patients admitted via the emergency department with subsequent intensive care treatment; patients who die before ICU admission should also be included. However, because the number of documented patients with only minor injuries has increased over time, the registry introduced a predefined ‘basic group’ that underpins most comparative analyses in the annual report. The TR-DGU basic group is defined as all patients with MAIS ≥3 plus MAIS 2 patients who died or required ICU treatment. Observational TR-DGU studies comparing helicopter EMS with ground EMS in the German setting (notably, both physician-staffed) consistently show that HEMS patients are more severely injured and receive longer and more intervention-intense prehospital care; after risk adjustment and/or propensity-score methods, helicopter transport has been associated with lower in-hospital mortality. Subgroup analyses further suggest that the association may be particularly relevant in older patients and in low-energy mechanisms such as low falls. While these trauma-center–anchored registry data are not directly interchangeable with our prehospital cohort, they offer an external benchmark and support the hypothesis that geriatric trauma dispatch criteria should integrate physiology and mechanism (including low-energy falls) alongside anticipated transport time/distance in order to mitigate under-triage (44–46).

The substantial trauma share in our cohort is consistent with Guirguis-Blake et al. (47). Falls are the leading cause of unintentional injury death in adults aged ≥65 years in the USA, with 714 falls per 1,000 older adults and 170 fall-related injuries per 1,000.

Our findings should be viewed through the lens of HEMS economics. Hungarian data estimate an operating cost of about EUR 2,488 per flight hour, which is about EUR 41.5 per minute in the air. When adjusted only for higher Western European wages, the estimate rises to about EUR 62.1 per minute. Personnel costs are the largest component. Pay-by-hour programs are also relevant, making costs largely variable. In our system most missions occurred in daytime, and single-mission duration often exceeded the sector reference of 20–40 min. Both factors modulate the daily cost of readiness and the case-level cost. Given our case mix with neurological and cardiovascular dominance and frequent advanced procedures, resource allocation by time of day and season could yield economic gains without harming clinical outcomes. We therefore recommend that Polish HEMS report standardized cost metrics per flight hour and per flight minute and a case-mix–adjusted cost. This would enable international comparisons and modeling of the impact of demographic aging on system financing (48).

### Limitations

The observed missions describe events at the prehospital stage. The analysis is retrospective and relies on operational and medical records, which introduces a risk of recording errors. Data do not cover in-hospital procedures provided to patients transported by HEMS. We cannot infer hospital course and outcomes, for example percutaneous coronary intervention (PCI) or thrombolysis, surgical procedures, or in-hospital mortality, nor the effectiveness of care across the full continuum. Access to medical documentation did not include hospital treatment.

A key limitation concerns endpoint definition and selection. Mortality was operationalized as on-scene death after HEMS arrival (left on scene - death) among missions with patient contact, while dispatches coded as DOA were excluded (Figure 1). Consequently, our estimates should not be interpreted as overall prehospital mortality after dispatch and do not capture deaths during transport or after hospital arrival. If DOA frequency differs systematically by age stratum, diagnosis, or dispatch pathway, exclusion of DOA may introduce selection (survivor) bias and could materially distort observed associations, including the counterintuitive lower on-scene mortality in the ≥75 group. In addition, the registry extract did not provide characteristics of excluded records (DOA, unknown patients, and records with missing key variables); therefore, we could not compare excluded versus included cases, which limits formal sensitivity analyses related to exclusions. In addition, the operational meaning of ‘on-scene death’ may differ between primary missions and IFT (referring facility vs. incident scene), so mission-type comparisons for mortality should be interpreted cautiously.

Age was analyzed in two strata (65–74 vs. ≥ 75 years) to maintain interpretability for operational stakeholders; however, we did not model age as a continuous predictor (or using flexible functions), which limits inference about dose–response patterns across the older age spectrum.

We also lacked data on comorbidity, chronic medication use, or Do Not Resuscitate (DNR) orders and palliative status. This limits our ability to account for key geriatric prognostic factors and increases the risk of residual confounding, particularly because regression analyses were unadjusted. The dataset does not identify the specific HEMS crew for each mission, so we did not analyze regional variation or compare mission intensity among patients ≥65 years across provinces. In addition, we did not include HEMS flights transporting blood, biological materials, or specialist staff for life-saving purposes in the studied age-defined cohort.

Times and distances were sourced from operational logs and may be subject to measurement error. Consolidation of diagnostic and pharmacologic categories simplifies analysis at the cost of clinical nuance at domain boundaries. Organizational changes during the decade, including opening new bases, dispatcher reorganization, and the COVID-19 period, may have influenced dispatch patterns and clinical reasons for calls.

We did not fit mission-type–specific main models nor formally test interaction with mission type. Therefore, residual heterogeneity by mission type may influence pooled unadjusted associations, and findings should be interpreted as descriptive rather than definitive comparative effects.

## Conclusion

HEMS in Poland plays a pivotal role in the care of older adults, especially in rural areas, where it enables rapid advanced procedures and direct hospital transport. The greatest clinical burden involves neurological and cardiovascular conditions, which differ in intervention profile. In the former, airway management and immobilization predominate. In the latter, resuscitation and electrotherapy are central. Seasonality is evident. Trauma rises in summer and respiratory disorders in winter. Together with the predominance of daytime missions, this should directly inform resource planning and team availability. We recommend refinement of dispatch criteria for low-energy mechanisms in seniors. Future prospective work and registry development could incorporate standardized geriatric measures (e.g., baseline functional status and, where feasible, CFS) to enable formal evaluation of their association with escalation decisions and outcomes. Counties with the highest geriatric load should be identified for targeted population interventions, including fall prevention, first aid education, and cardiovascular prevention. These measures can improve outcomes and system efficiency in an aging population.

This nationwide analysis provides the most comprehensive characterization to date of HEMS operations in Poland among older adults. The results highlight both the strengths and the evolving challenges of prehospital care in an aging society. HEMS clearly fulfills a compensatory role for regional inequalities by ensuring access to time-critical interventions across large rural areas. However, the findings also underscore the need for modernization of geriatric-sensitive dispatch algorithms and for integration of geriatric-sensitive decision support and standardized data capture. The lower rates of invasive procedures and on-scene mortality after HEMS arrival among patients aged ≥75 years may reflect a complex balance between case-mix, illness severity, baseline vulnerability, and ethical considerations surrounding intervention intensity; however, frailty was not measured and our analyses were unadjusted, so frailty-specific interpretations remain speculative. Strategically, the study suggests that demographic aging will continue to shift HEMS demand toward neurological and cardiovascular emergencies, with pronounced seasonal variability. These trends should guide future workforce planning, training in geriatric prehospital medicine, and resource allocation to maintain readiness for daytime and seasonal peaks. Future research should link prehospital data with in-hospital outcomes to evaluate the full continuum of geriatric emergency care and to assess how frailty and polypharmacy affect survival and recovery trajectories.

In conclusion, adapting HEMS systems to the realities of population aging requires both operational and conceptual innovation. Refining age- and mechanism-adjusted dispatch criteria and integrating prehospital and hospital data registries may enhance equity, efficiency, and outcomes for older adults. If frailty-related hypotheses are to be tested, standardized frailty measurement should be incorporated prospectively rather than inferred from age or clinical presentation.

## Data Availability

The datasets presented in this study can be found in online repositories. The names of the repository/repositories and accession number(s) can be found in the article/supplementary material.
